# Cigarette Smoke Impairs A_2A_ Adenosine Receptor Mediated Wound Repair through Up-regulation of Duox-1 Expression

**DOI:** 10.1038/srep44405

**Published:** 2017-03-24

**Authors:** Zhi Tian, Hui Zhang, Jendayi Dixon, Nicole Traphagen, Todd A. Wyatt, Kusum Kharbanda, Samantha Simet Chadwick, Narasaiah Kolliputi, Diane S. Allen-Gipson

**Affiliations:** 1 Department of Pharmaceutical Sciences, College of Pharmacy, University of South Florida, Tampa FL, USA; 2 Division of Pulmonary, Critical Care, Sleep and Allergy, Department of Internal Medicine, University of Nebraska Medical Center, Omaha, NE, USA; 3 Department of Environmental, Agricultural, and Occupational Health, College of Public Health, University of Nebraska Medical Center, Omaha, NE, USA; 4 Research Service, Omaha-Western Iowa Veterans Affairs Medical Center, Omaha, NE, USA; 5 Division of Gastroenterology and Hepatology, Department of Internal Medicine, University of Nebraska Medical Center, NE, USA; 6 Division of Allergy and Immunology, Department of Internal Medicine, College of Medicine, University of South Florida, Tampa FL, USA.

## Abstract

Cigarette smoke (CS) exposure and intrinsic factors such as the NADPH oxidases produce high levels of reactive oxygen species (ROS), ensuing inflammatory tissue injury. We previously demonstrated that CS-generated ROS, particularly hydrogen peroxide (H_2_O_2_), impaired adenosine stimulated wound repair. We hypothesized that CS exposure modulates expression of Dual oxidase 1 (Duox-1), a NADPH oxidases known to generate H_2_O_2_. To test this hypothesis, we used human bronchial epithelial cell line Nuli-1 and C57BL/6 mice. Cells were treated with 5% CS extract (CSE) for various periods of time, and mice were exposed to whole body CS for six weeks. Both CSE and CS treatment induced increased expression of Duox-1, and silencing of Doux-1 improved the rate of cell wound repair induced by CSE treatment. Nuli-1 cells pretreated with thapsigargin but not calcium ionophore exhibited increased Duox-1 mRNA expression. CSE treatment stimulated PKCα activation, which was effectively blocked by pretreatment with diphenylene iodonium, a NADPH oxidase inhibitor. Compared to control, lungs from CS-exposed mice showed a significant increase in PKCα activity and Duox-1 expression. Collectively, the data demonstrated that CS exposure upregulates expression of Duox-1 protein. This further leads to H_2_O_2_ production and PKCα activation, inhibiting A_2A_AR-stimulated wound repair.

We previously demonstrated that CS-generated ROS, particularly H_2_O_2_, is implicated in blunting ADO-mediated wound repair of airway epithelial cells^1^,^2^. However, the mechanism by which CS-generated H_2_O_2_ contributes to the dysregulation of ADO-stimulated wound repair remains unknown. CS exposure produces high levels of ROS, and this increase has been linked to the activation of the Nox enzyme family NADPH oxidases^3^,^4^. The biological function of NADPH oxidases is to generate ROS, and excessive ROS production has been associated with inflammatory tissue injury^4^,^5^. Within the NADPH enzyme family, Dual oxidase 1 and 2 (Duox-1, Duox-2) are known to generate H_2_O_2_ either directly or indirectly by rapid dismutation of superoxide^6^. The objective of this study was to determine whether CS exposure of airway epithelial cells activates signaling pathways associated the Duox-1 and 2 generation of H_2_O_2_.

Many potential signaling pathways are associated with ADO-mediated effects. The prime candidate signaling systems includes cyclic nucleotides and their target kinases. Cyclic nucleotides such as cAMP and cGMP are key messengers in modulating cell shape and attachment as well as cell movement in many model systems^7^,^8^. We recently demonstrated that CS exposure blunts ADO-mediated activation of cAMP-dependent Protein Kinase A (PKA) and this was facilitated by the robust activation of PKC signaling^2^. Moreover, we showed previously that PKC activation retards wound repair^9^. To accomplish our objective, we hypothesized that CS exposure activates Duox-1 and/or 2, and the subsequent generation of H_2_O_2_ activates PKC, which modulates ADO’s airway epithelial cell repair and recovery.

## Results

### CSE and CSE-Generated Oxidants Impair A_2A_ Receptor (A_2A_AR)-mediated Airway Epithelial Cell Wound Repair

**Figure 1:**
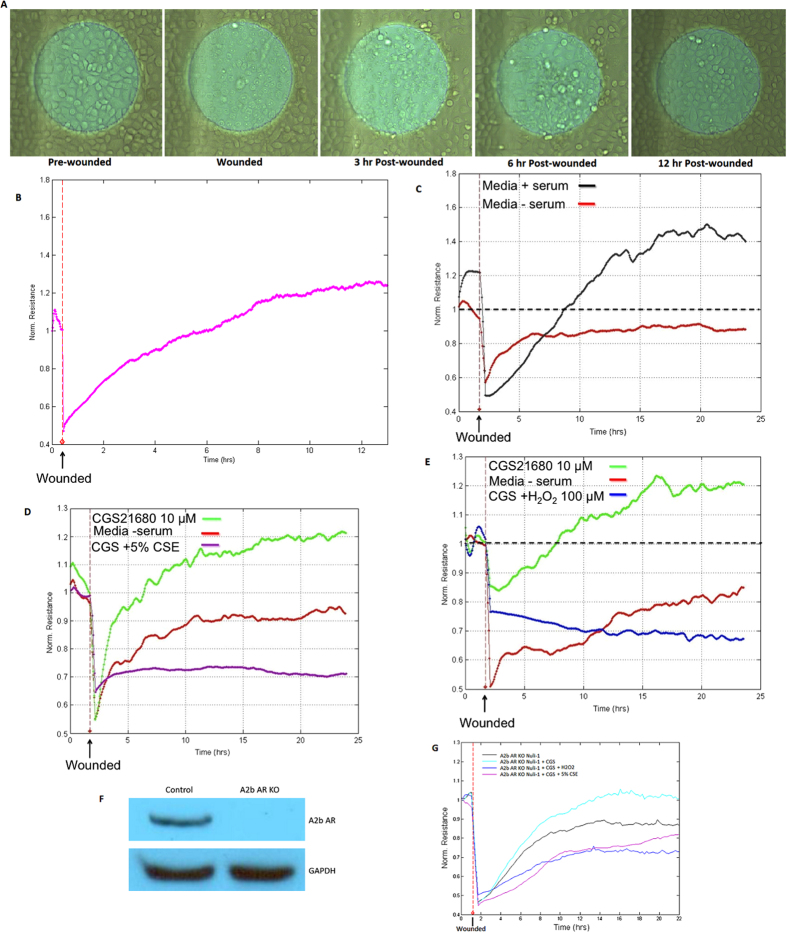
Injury and Repair of Nuli-1 Human Epithelial Cells. Photomicrographs of cell monolayers (**A**) and changes in TEER (**B**) during cell migration after wound (arrow). (**C**) Serum stimulation accelerates and enhanced wound-healing capacity of Nuli-1 cells. Each condition was conducted in triplicate and a representative of one experiment repeated at least two times is shown. (**D**) Stimulated A_2A_AR with CGS21680 improved wound healing. CSE treatment inhibits A_2A_AR-stimulated wound closure. Each condition was conducted in duplicate and is representative of an experiment repeated at least two times. (**E**) H_2_O_2_ exposure blocks the enhanced wound closure mediated by activated A_2A_AR. Each condition was conducted in duplicate and is representative of an experiment repeated at least two separate times. (**F**) Efficient A_2B_AR knockout in Nuli-1 cells. (**G**) Both CSE and H_2_O_2_ inhibit A_2A_AR-stimulated wound closure in A_2B_AR knockout Nuli-1 cells.

We used the ECIS system for evaluating cell wound repair because it generates consistent size of injury (250 μm diameter) and produces quantifiable, automated, real time data. Upon cell monolayer wounding, repair is initiated with cell migration resulting in a rise in TEER as observed in Fig. 1A and B. Repair is mostly accomplished around 12 h post-injury. Serum stimulation accelerates the rate of recovery (Fig. 1C) and serves as positive control. Nuli-1 cells are A_2A_AR and A_2B_AR dominant cells (Supp 1). To determine the better concentration to activate A_2A_AR, Nuli-1 cells were treated with 10 μM, 100 nM or 10 nM CGS21680, a potent and selective A_2A_AR agonist, for 24 h. The expression level of A_2A_AR in cells treated with 10 μM CGS21680 is significantly higher than 10 nM treated group, and observed the expression level of A_1_AR lower than 10 nM treated group (Supp 2). Stimulation of cells with CGS21680 10 μM improves the rate of repair (Fig. 1D), indicating that A_2A_AR activation mediates airway epithelial cell wound repair. However, the A_2A_AR-mediated wound closure effect was abolished by CSE exposure (Fig. 1D). CSE-treated cells exhibited no significant release of lactate dehydrogenase compared to no-CSE treated cells (data not shown). To correlate the effect of CSE-generated oxidants on A_2A_AR-mediated wound closure ability, cells were exposed to H_2_O_2_ 100 μM, electrically wounded, and stimulated with CGS21680. Cells exposed to H_2_O_2_ and stimulated with CGS21680 had a marked decrease in TEER compared to the CGS21680 alone group (Fig. 1E). Interestingly, lower concentration of H_2_O_2_ (1–10 μM) had no effect in inhibiting CGS21680-stimulated wound closure (data not shown). Because micromolar concentration of CGS21680 may also activate A_2B_AR, experiments were repeated using A_2B_AR knockout Nuli-1 cells (Fig. 1F) treated with CSE or H_2_O_2_, in the presence or absence of CGS21680 stimulation. Figure 1G shows enhanced wound closure in A_2B_AR knockout cells stimulated with CGS21680, which is consistent with the role of A_2A_AR in repair after injury. Pre-exposure of A_2B_AR knock-out Nuli-1 cells stimulated with CGS21680 to CSE or H_2_O_2_, decreased TEER compared to non-pre-exposed cells implying that A_2A_AR but not A_2B_AR is the target of inhibitory effect. These findings suggest that the balance of oxidants/antioxidants is central in the regulation of ADO-mediated repair processes and that CSE-generated oxidants play a role in impairing A_2A_AR-mediated epithelial cell repair after injury.

### Hydrogen Peroxide Activates PKC in Bronchial Epithelial Cells

**Figure 2:**
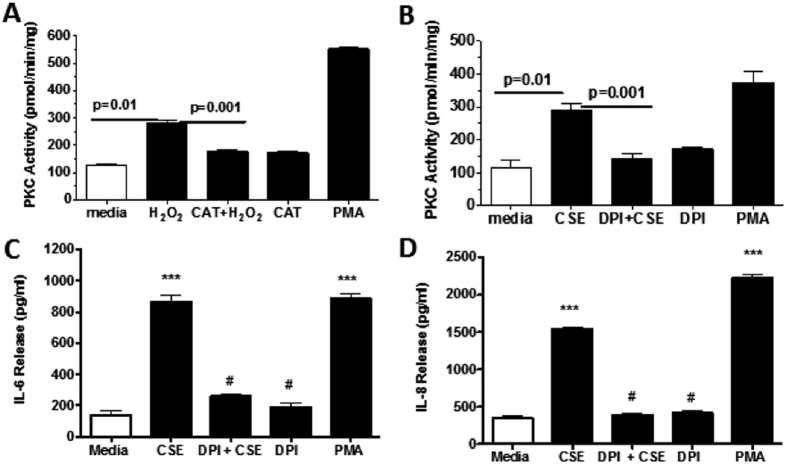
(**A**) Activation of PKC by hydrogen peroxide administration. Nuli-1 cells were pretreated 1 h with or without catalase (500 U/mL), and then wounded and stimulated with (black bars) or without CSE (white bars). Data are mean ± SE of three separate experiments done each in triplicate. (**B**) NADPH oxidase inhibition abrogates CSE-stimulated PKC activation. Nuli-1 cells were pretreated for 1 h with 1 μM of DPI, and then treated as above. DPI significantly blunted CSE-mediated activation of PKC. Data are mean ± SE of triplicate wells within a single experiment. (**C**,**D**) NADPH oxidase inhibition blunts CSE-mediated secretion of IL-6 and IL-8. Supernatants were collected from Nuli-1 cells pretreated with DPI as described above, and secreted IL-6 and IL-8 measured. DPI blunted CSE-mediated secretion of IL-6 and IL-8. Data are representative of one experiment repeated on three separate.

We reported that ADO occupancy of A_2A_AR stimulated wound closure in epithelial cells, and that this effect was mediated through cAMP-dependent kinase PKA pathway^10^. On the other hand, agents that activate protein kinase C (PKC), such as CSE and malondialdehyde-acetaldehyde adducted protein^9^,^11^, slowed cell migration in wound repair. Oxidative conditions have been demonstrated to selectively modify the regulatory domain of PKC, resulting in persistent active kinase^12^,^13^. To elucidate the mechanism of impaired A_2A_AR-mediated wound healing by CSE and H_2_O_2_ in epithelial cells, we investigated the involvement of PKC and oxidant species metabolizing enzymes. Wounded Nuli-1 cells were exposed to H_2_O_2_ (1 μM, a non-cytotoxic concentration), and PKC activity assessed. Some cells were pretreated with catalase (500 U/mL), an enzyme that primarily degrades H_2_O_2_, or with phorbol myristate acetate (PMA, a PKC activator, 100 nM; used as positive control). H_2_O_2_ significantly enhanced PKC activation; this response was blocked in cells pretreated with catalase (Fig. 2A). Because the precise mechanism involved in kinase activation in response to production of H_2_O_2_ in airway epithelial cells is not fully understood, we explored the potential role of NADPH oxidases in CSE-stimulated PKC activation. Wounded bronchial epithelial cells were exposed to CSE, and PKC activity quantified; some cells were pretreated with DPI, a NADPH oxidase inhibitor (1 μM), or with PMA. Figure 2B shows that CSE exposure significantly increased PKC activity. However, pretreatment with DPI markedly reduced CSE-stimulated PKC activation compared to CSE-treated cells. This inhibition of CSE-mediated PKC activation by DPI suggests that Duox oxidase enzymes are present in the airway epithelium.

CSE treatment induces an inflammatory environment. We investigated the role of NADPH oxidase in CSE-mediated release of the pro-inflammatory cytokines IL-6 and IL-8. CSE significantly enhanced both IL-6 and IL-8 release from epithelial cells (Fig. 2C and D; ***P < 0.001). However, pretreatment with DPI significantly reduced CSE-mediated release of IL-6 and IL-8 (^#^P < 0.001). Collectively, the data suggests that NADPH oxidases mediate PKC activation and promote secretion of pro-inflammatory cytokines. This provides evidence that CSE-generated oxidants may further potentiate CSE-mediated inhibition of A_2A_AR-stimulated wound repair.

### CSE Modulates Duox-1 Expression in Nuli-1 Cells

**Figure 3:**
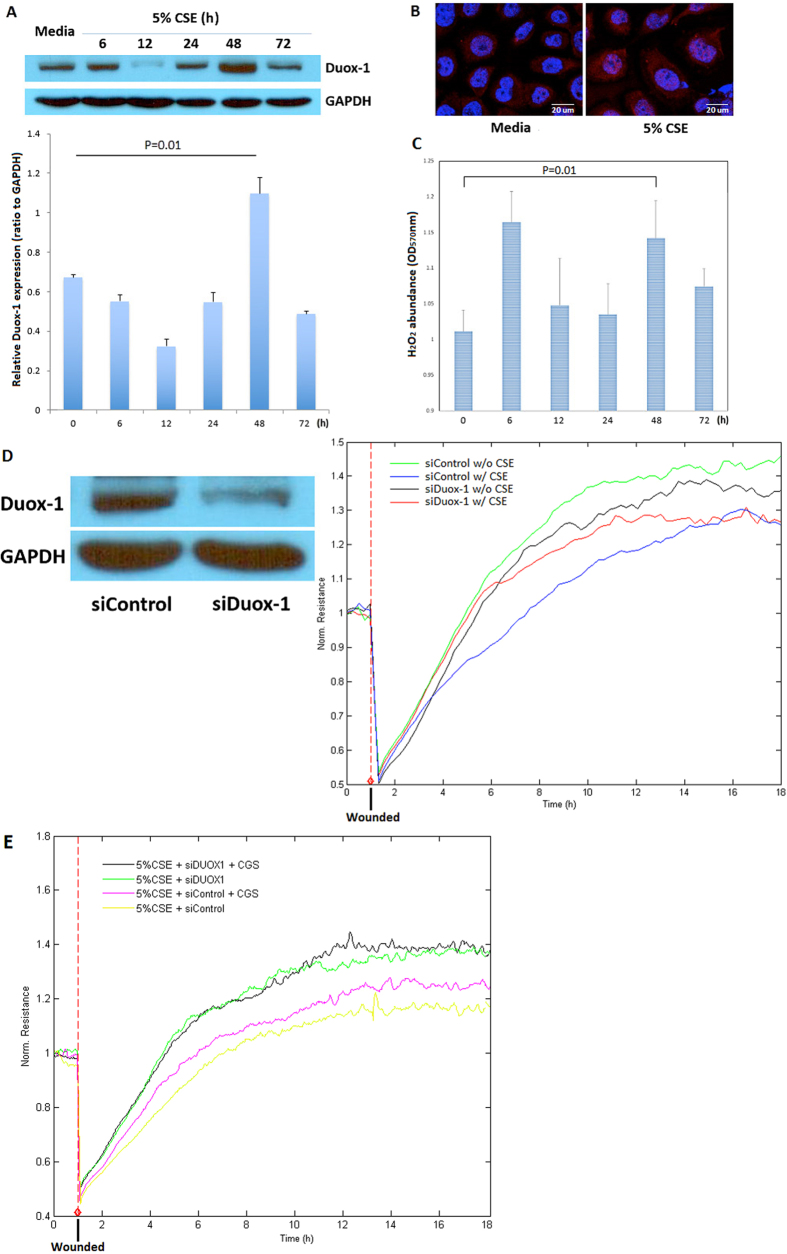
(**A**) CSE stimulates Duox-1 protein expression. Representative western blot of total cell lysates identifies a single band at 177 kDa of Duox-1. Peak of Duox-1 expression in cells treated with CSE is maximally by 48 h (graph). (**B**) Increase Duox-1 abundance in intact epithelial cells. Nuli-1 cells were exposed to 5% CSE for 48 h and Duox-1 visualized using a specific antibody followed by secondary antibody conjugated to Alex Fluor 647 (red); nuclear staining with DAPI (blue). Images are representative of at least four independent experiments. (**C**) CSE Exposure generates H_2_O_2_ in Nuli-1- cells. Nuli-1 cells were exposed to CSE for the indicated time periods, and H_2_O_2_ measured in the supernatants. (**D**) Duox-1 silencing ameliorates CSE-mediated inhibition of Nuli-1 cellular migration. 2 × 10^6^ cells were transfected with siControl or siDuox-1 siRNAs. After 48 h, cells were exposed to CSE for another 48 h, and wounds created. Resistance was measured in duplicate wells. A representative experiment is shown of two independently performed. (**E**) Duox-1 silencing impairs CGS21680 improved inhibition of CSE-treated Nuli-1 cellular migration.

It is well established that CSE exposure induces high levels of ROS formation, which has been linked to the activation of the Nox family of NADPH oxidases^3^,^4^. Duox-1 and 2 belong to the NADPH oxidases family and are known to generate H_2_O_2_ either directly or indirectly by dismutation of superoxide^6^. To further understand the mechanism associated with the deregulation of ADO airway epithelial wound repair, we investigated the effect of CSE on Duox expression and function in the airway epithelial cells. Cells were treated with CSE for 6, 12, 24, 48 or 72 h, and Duox-1 protein expression evaluated. CSE significantly increased Duox-1 abundance at 48 h (Fig. 3A). Duox-2 showed a weaker signal than Duox-1 when exposed to CSE (not shown), suggesting that the predominant Duox protein in the airway epithelium induced by CSE treatment is Duox-1. Figure 3B confirms enhanced abundance of Duox-1 in the cytoplasm of human Nuli-1 cells exposed to CSE. Consistently, H_2_O_2_ levels also increased in Nuli-1 cells treated with CSE for 48 h, as compared to untreated Nuli-1 cells (Fig. 3C); note an early peak of H_2_O_2_ production at 6 h of CSE exposure. To determine whether Duox-1 function plays an essential role in ADO airway epithelial wound repair, we silenced Duox-1 expression (siDuox-1 cells) and examined the effect of decreased Duox-1 abundance on cell migration. Compared to siControl cells treated with CSE, the rate of TEER elevation is preserved in siDuox-1 cells exposed to CSE and it is similar to that in untreated cells (Fig. 3D). Moreover, stimulation of 5% CSE treated cells with CGS21680 improves the rate of repair; but in Duox-1 silenced Nuli-1 cells, CGS21680 treatment had no significant effect in increasing CSE-stimulated wound closure (Fig. 3E). Collectively, these results demonstrated that CSE stimulates expression of Duox-1, which is a major mediator of airway epithelial cell H_2_O_2_ production and of impaired repair after cell injury.

### Increased Levels of Intracellular Calcium Upregulate Duox-1 Expression and are Induced by CSE Treatment

**Figure 4:**
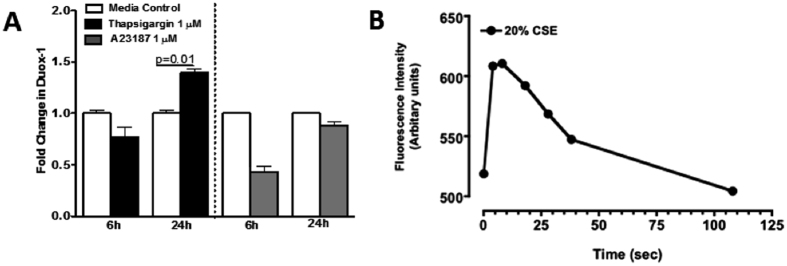
(**A**) Elevation of cell [Ca^2+^]_i_ upregulates Duox-1 transcriptional expression. Duox-1 mRNA levels in Nuli-1 cells treated with thapsigargin (black bars) or A23187 (grey bars) were quantified using Taqman PCR. Values were normalized to 18S rRNA, and results expressed as mean fold change from control cells ± SE of three experiments each performed in duplicate. (**B**) Incubation with 20% CSE stimulates Ca^++^ mobilization.

Duox-1 and Duox-2 contain EF motifs calcium-binding domain that can be directly activated by Ca^++^ 14,15. To examine the effect of calcium on Duox-1 expression, Nuli-1 cells were treated for 6 or 24 h with thapsigargin (1 μM, a calcium pump blocker that inhibits re-entry of cytosolic calcium into the sarcoplasmic reticulum) or with the calcium ionophore A23187 (1 μM). After 24 h, thapsigargin significantly increased Duox-1 mRNA abundance (P = 0.01) while A23187 treatment had no effect at either time point (Fig. 4A). This demonstrates that intracellular calcium mobilization stimulates Duox-1 gene expression. Treatment with 20% CSE induced a slower and transient increase of intracellular calcium concentration ([Ca^++^]i) before returning to baseline levels (Fig. 4B). Nuli-1 cells stimulated with ATP (1 μM; positive control) exhibited a rapid transient increase of [Ca^++^]i (data not shown). These studies suggest that increased intracellular calcium mobilization caused by CSE exposure may mediate Duox-1 activation.

### Chronic CS Exposure Induces Inflammation, Increases PKC Activity, and Upregulates Duox-1 Expression in Mice

**Figure 5:**
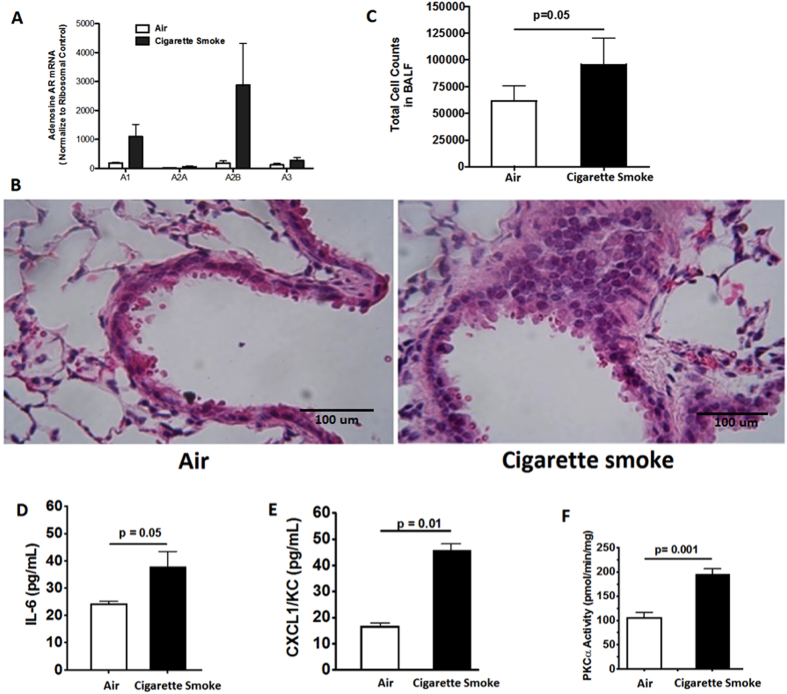
(**A**) Effect of CS on transcript levels of A_1_AR, A_2A_AR, A_2B_AR, and A_3_AR. Expression of mRNA in lung homogenates of mice treated with air of CS for 6 weeks. Values are mean ± SE from 3–4 animals per group. (**B**) Lung histology. Mice exposed to CS show airway abnormalities. Images are representative from 6-8 animals analyzed per group. (**C**) Chronic CS exposure increases the number of BAL cells in mice. Values are expressed as mean ± SE from 6-8 animals per group. P = 0.05 vs. mice exposed to air alone. (**D**,**E**) IL-6 and CLCX1/KC levels are increased in the BAL fluid of mice exposed to CS. Values represent mean ± SE of IL-6 or CXCL1/KC from 6–8 animals per group. (**F**) CS exposure increases activity of PKCα. Values represent the mean ± SE of PKC activity in lung tissues from 6–8 animals per group.

**Figure 6:**
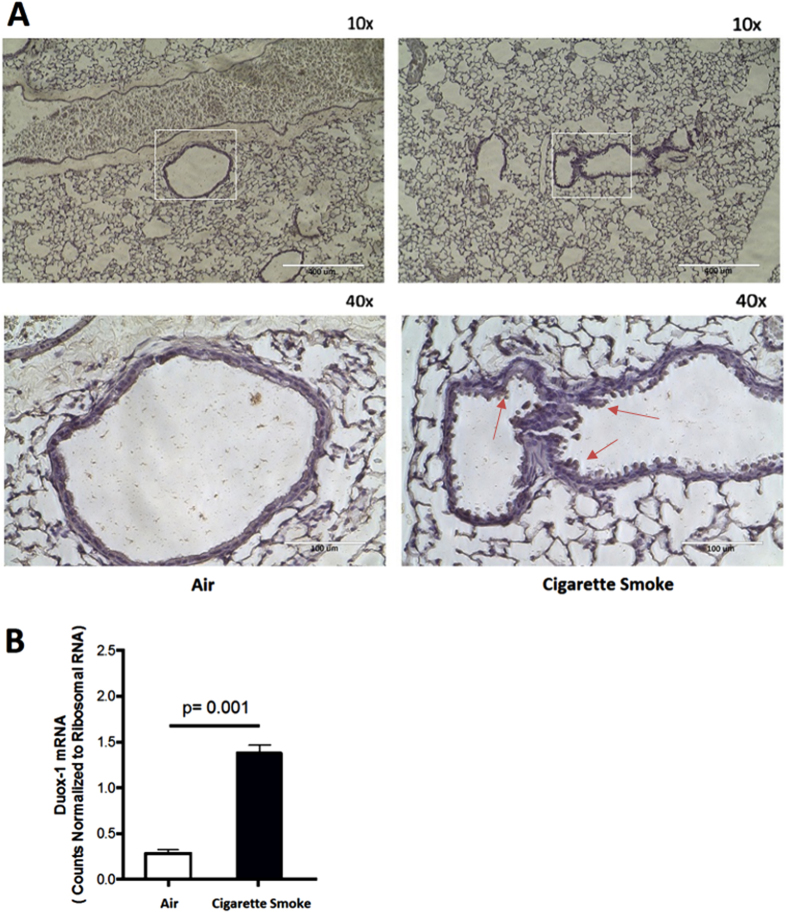
(**A**) Whole body CS exposure upregulates Duox-1 expression. (**A**) Lung immunohistochemistry. (**B)** Transcript levels of Duox-1 were normalized to 18 S rRNA.

We studied the effect of CS as a model of airway epithelium damage to elucidate the CS-oxidant driven mechanism(s) in mice exposed to whole body CS for six weeks. Real-time qPCR analysis shows no significant differences in total lung A_2A_AR mRNA expression between the air-treated and CS-treated animals (Fig. 5A). Lung pathology revealed increased inflammatory cells and hypertrophy of the bronchial airway epithelium in CS-exposed mice compared to air exposed controls (Fig. 5B). Mice treated with CS showed a significant increase in the total number of BAL cells compared to control animals (Fig. 5C). In addition, IL-6 and CXCL1/KC amount in BAL fluid from CS-treated group was significantly elevated compared to the air group (Fig. 5D and 5E; IL-6, P = 0.05; KC, P = 0.01). PKC activity in the CS-treated group as compared to the control group (Fig. 5F; P = 0.001). Finally, immunohistochemical analysis showed Duox-1 expression in tracheobronchial epithelial cells of air-treated control mice, while the CS-treated group had a marked increase Duox-1 cellular localization (Fig. 6A). RT-PCR also revealed augmented Duox-1 transcriptional levels in the CS treated-group compared to controls (Fig. 6B, P = 0.001). Together, these results demonstrate that elevation of pro-inflammatory cytokine secretion, PKC activity, and Duox-1 expression observed in human cultured epithelial cells treated with CSE are recapitulated in mice exposed chronically to CS. We speculate that CS-mediated increase of Duox-1 may further lead to H_2_O_2_ production and deregulation of ADO-mediated repair of the airway epithelium.

## Discussion

**Figure 7:**
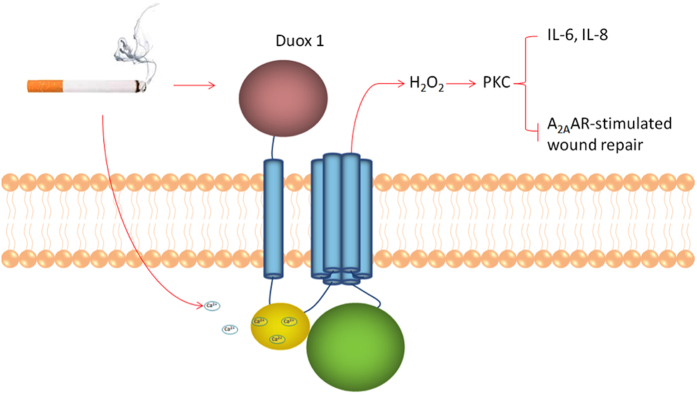
Schematic representation of the role of Duox-1 in CS impairment of A_2A_AR mediated wound repair in airway epithelial cells.

CS is a major risk factor for a number of chronic respiratory diseases including asthma and chronic obstructive pulmonary disease (COPD). COPD is the third leading cause of mortality and morbidity in the United States (CDC Report, 2010), and is characterized by persistent inflammation and injury of both the airways and the parenchyma of the lung. CS exposure has been associated with high levels of oxidative stress and is involved in many biological processes, such as inflammation and carcinogenesis^16^,^17^. We previously demonstrated that ADO acts via the A_2A_AR to promote repair after wound injury in bronchial epithelial cells^10^,^18^. Recently, we implicated CS-generated reactive oxidants in the CS impairment of ADO-mediated wound healing^2^. The present study was designed to further understand the mechanism underlying CS generation of H_2_O_2_ and the dysregulation of ADO-stimulated wound repair of airway epithelial cells. We found that CS exposure induced cell calcium mobilization, upregulated mRNA and protein expression of the NADPH oxidase Duox-1 in human cells and mice lungs, and promoted Duox-1 activation. This further leads to H_2_O_2_ production, with subsequent PKC activation and promotion of pro-inflammatory cytokines secretion and inhibition of A_2A_AR-stimulated wound repair (Fig. 7).

The A_2A_ARs can couple to both PKA and PKC dependent downstream cascades and these two transducing systems can crosstalk. Our earlier studies showed that stimulation of PKC blunted A_2A_AR-mediated PKA activation^2^, that PKC activation retards wound repair^19^,^20^, and that exposure to CSE increases PKC activity^2^. In this study, we demonstrated that PKC activity is increased in mice expose to CS. Collectively, our findings suggest that CS inhibition of A_2A_AR-mediated activation of PKA is mediated by activation of PKC both *in vitro* and *in vivo*.

We previously demonstrated that CS-generated H_2_O_2_ is implicated in blunting ADO wound repair^1^,^2^. In this study, we observed that both CSE and H_2_O_2_ significantly enhanced PKC activation, and that CSE stimulated the release of IL-6 and IL-8 in human cultured airway epithelial cells. These findings support our earlier studies in which CS-mediated IL-8 release blunted ciliary motility (a first line of defense in the airway)^21^ and activation of PKC required CS-enhanced C5a-mediated release of IL-8^22^. In this study we observed that pretreating cells with DPI or catalase, prevented PKC activation, suggesting that H_2_O_2_ and intrinsic factors such as dual oxidases may play a pivotal role in the dysfunction of ADO-mediated wound repair.

CS can induce intrinsic free radical production, which, through Duox-1 and 2 action, is responsible for increased airway H_2_O_2_ generation^6^,^15^. We showed that silencing expression of Duox-1 rendered epithelial cells partially resistant to the negative effect of CSE on wound healing after injury. Little is known about the regulation of dual oxidases in association with smoking and/or COPD. Lavigne *et al*., reported that Duox-1 mRNA expression did not change after NHBE cells were treated with CSE for 60 min^23^, and Nagai *et al*., reported that Duox-1 expression in tracheal and bronchial epithelial brushings was significantly down-regulated, although Duox-2 was up-regulated, in current smokers compared to individuals who never smoked^24^. We observed that expression of Duox-1 was temporally regulated by CS exposure (Fig. 3A) and evident in the lower airway of mice (Fig. 6A). Thus, Duox-1 mRNA expression was down-regulated within 12–24 h by CS, but after 48 h the expression of Duox-1 significantly increased, and by 72 h it was further down-regulated. This finding suggests a temporally differential effect of CS on Duox-1 expression, indicating that the observation from Lavigne’s group was likely due to acute CS exposure, and it is consistent with Nagai’s report on Duox 1 down-regulation in human tissue from current smokers.

Figure 7 summarizes our results on the proposed role of CS on Duox-1 expression and function. During acute exposure, CS increases intracellular calcium mobilization to activate Duox-1. The activation of Duox-1 catalyzes the dismutation of superoxide to H_2_O_2_. The increased amounts of H_2_O_2_ stimulate PKC activation and promote the release of pro-inflammatory cytokines, which eventually inhibits A_2A_AR-stimulated wound repair. Our future studies plan to use Doux-1 knockout mice to further establish the intersection between Duox-1 with A_2A_AR signaling transduction and whether there is an additional pathway that alters A_2A_AR-mediated airway wound repair in chronic respiratory diseases.

## Material and Methods

### Cell Preparation

The Nuli-1 human bronchial epithelial cell line was purchased from the American Type Culture Collection (Rockville, MD). Cells were cultured on type VI placenta collagen (Sigma, St. Louis, MA) coated dishes in serum-free bronchial epithelial growth media (BEGM; Lonza, Walkersville MD). Cells were maintained in culture at 37 °C in humidified 95% air −5% CO_2_.

### Animals

C57BL/6 mice were purchased from Charles River Laboratories (Wilmington, MA) at 8 weeks of age and maintained under standard housing conditions in the animal care facility at the University of Nebraska Medical Center (UNMC) and University of South Florida (USF); the American Association of Accreditation of Laboratory Animal Care (AAALAC) accredits both institutions. Mice were acclimated to the facility for one week prior to the start of exposure and received water and standard rodent chow *ad libitum* for the entire course of the study. All studies were carried out in accordance with the *Guide for the Care and Use of Laboratory Animals of the National Institutes of Health* and were approved by the UNMC and USF Institutional Animal Care and Use Committees.

### CS and Air Exposure

Mice were passively treated with CS using a whole-body smoke exposure system (Teague Industries, Davis, CA). Using the Teague device^25^, mice were exposed to smoke from sixty **3R4F** reference cigarettes (University of Kentucky, Lexington, KY) per day. Mice receiving CS were gradually brought to their target exposure over a period of seven days, treated 5 days/week for 6 weeks. Control mice were exposed to air in the same manner in a similar apparatus for the same periods of time.

### Cigarette Smoke Extract (CSE) Preparation

CSE was prepared as previously described^26^ using **3R4F** reference cigarettes (University of Kentucky, Lexington, KY).

### Bronchoalveolar Lavage Fluid (BALF)

Mice were euthanized by intraperitoneal injection of 75 mg/kg sodium pentobarbital (Nembutal; Abbott Labs, Chicago, IL) as previously described by Elliott *et al*.^27^. Tracheas were exposed, nicked at the bottom of the larynx and cannulated. The proximal ends of the tracheas were tied off and 1.0 mL of cold sterile PBS (Gibco, Grand Island, NY) was gently flushed into the lungs and recovered; a total of three washes were performed and combined. Collected BALF was centrifuged at 300 g for 7 min at 4 °C. The supernatant was stored at −80 °C until use for measurement of cytokines concentration. Pelleted cells were resuspended in 1.0 ml of PBS. Total cell were counted on a hemocytometer, and 1–5 × 10^3^ cells were spun onto glass microscope slides (cytospin 3; Shandon Scientific, Cheshire, UK). Cells were air dried for 24–36 h, fixed, and stained with a Diff-Quik stain set (Dade Behring, Newark, DE). Differential cell counts of at least 300 cells per slide were made according to morphological criteria. The number of cells recovered was calculated and expressed as absolute cell numbers.

### Lung Collection and Histology

Whole lungs were excised and inflated to 10 cm H_2_O pressure with 10% formalin (Sigma, St. Louis, MO) to preserve pulmonary architecture. Lungs were embedded in paraffin, and sections (4–5 μm) were cut and processed for hematoxylin and eosin staining.

### Immunohistochemistry and Immunofluorescence

Lung sections were deparaffinized in xylene and rehydrated, and antigen retrieval was performed in PBS containing 0.1% trypsin for 1 h in a humidified chamber. Goat anti Duox-1 (1:500) IgG antibody was from Abcam (Cambridge, USA). Vectastain ABC kit was used to detect Doux-1 with diaminobenzidine (DAB; brown) and counterstained with hematoxylin control samples were blocked with 10% normal goat serum. Cells were seeded at a density of 20000 cells/cm^2^ on collagen-coated coverslips overnight and fixed with 4% paraformaldehyde (Rockford, USA) for 15 min. The cells were blocked for 30 min with antibody diluting buffer (1.5% goat serum in PBS with 0.3% Triton X-100) and incubated with Duox-1 antibody (Abcam, Cambridge, USA) at 1:100 dilution overnight. Cells were incubated with Alex Fluor 594-CONJUGATED goat anti-rabbit IgG (1:100) (Life technologies, Eugene, USA) for 30 min. An extensive wash was performed between each step. The cells were then mounted with VECTASHIELD (Vector, Burlingame, USA) and imaged using an Olympus 3i Spinning Disk Confocal Microscope (Olympus, Center valley, PA).

### PKC Activity Assay

PKC activity was determined as previously described^28^ using a reaction solution containing 24 μg/ml PMA, 30 mM dithiotreitol, 150 μM ATP, 45 mM Mg-acetate, PKC isoform-specific substrate peptide (Santa Cruz, Dallas, USA), and 10 μCi/mL [γ-^32^P]-ATP Tris-HCl buffer (pH 7.5). Samples (20 μl) were added to 40 μl of the reaction solution and incubated for 15 min at 30 °C. This mixture (60 μl) was then spotted onto P-81 phosphocellulose paper (Whatman, Clinton, NJ) to halt incubation. Immediately, papers were washed five times with 75 mM phosphoric acid for 5 min, once with 100% ethanol for 1 min, dried, and radioactivity counted in nonaqueous scintillant (National Diagnostics, Atlanta GA). Negative controls were carried out in similar assay conditions but without substrate. PKC activity was normalized to total protein assayed and expressed in picomoles of phosphate incorporated per minute per milligram of total protein. All samples were assayed in triplicate.

### Electric Cell Substrate Impedance Sensing (ECIS) Wounding (Migration) Assay

Cell resistance was measured using the electric cell–substrate impedance sensing system (ECIS; Applied BioPhysics, Troy, NY). Cells are cultured onto gold electrodes and impedance values recorded, which are transformed to both resistance and capacitance values. As cells grow and become confluent, they constrict current flow through the electrodes and alter impedance^29^. Nuli-1 cells were grown to confluence on ECIS 96-well plate arrays (8W1E; Applied Biophysics, Troy NY). Cell monolayer were wounded using an elevated field pulse of 1400 μA at 32,000 Hz applied for 20 sec, producing a uniform circular lesion 250 μm in size. The wounds were tracked over a period of 24 h. The impedance was measured at 4000 Hz, and the transepithelial resistance (TEER; ohms) normalized relative to the value at the start of data acquisition previous to treatments, and plotted as a function of time.

### Cytokines Analysis

IL-6 and IL-8 concentration in Nuli-1 cells exposed to CSE or air in the presence or absence of the NADPH oxidase inhibitor DPI was determined using Quantikine ELISA kits (R&D Systems, Minneapolis, MN). Similarly, IL-6 and CXCXI/KC levels in BALFs collected from mice exposed to CS or air were determined using Quantikine ELISA kits (R&D Systems, Minneapolis, MN). Samples were incubated for 2 h and washed with Quantikine wash buffer as per manufacture instruction. Absorbance was measured at 450 nm with a 540 nm correction, and concentrations calculated per the manufacturer’s instructions.

### Taqman Real-time RT-PCR

RNA was extracted using the Magmax 96 kit (Applied Biosystems, Foster City, CA) according to the manufacturer’s instructions. RNA concentration and purity was determined with NanoDrop spectrophotometer. cDNA was synthesized by using 100 ng of total RNA and TaqMan reverse transcription kit (Applied Biosystems, Foster City, CA). Real-time PCR reactions were prepared in triplicate using 1X TaqMan Master Mix (Applied Biosystems) and primers and probes, as previously described^18^. 18S ribosomal RNA was used as endogenous control. qPCR was performed using an ABI PRISM 7700 Sequence Detection System (Applied Biosystems). Threshold values (Ct) were normalized to that of 18S rRNA. Results were expressed either as the percent increase in induction (100× normalized values of stimulated cells divided by normalized values of unstimulated cells) or as values normalized to expression of rRNA.

### siRNA

Accell^TM^-siRNAs were purchased from Dharmacon (GE Healthcare Dharmacon Inc., US). Nuli-1 cells were plated at a density of 30–40% confluency and incubated overnight. Accell Duox-1 siRNA or non-targeting siRNA was added to final concentration of 1 μM. Transfected cells were incubated for 96 h before treatment.

### Generation of A_2B_ Adenosine Receptor (A_2B_AR) Knock-out Nuli-1 Cells

A_2B_AR knockout Nuli-1 cells were generated using CRISPR/Cas9 system according to Ran’s protocol^30^. Briefly, guide RNA (gRNA) for A_2B_AR was designed using an online tool (http://www.e-crisp.org/E-CRISP/designcrispr.html), (5′-GCTGGTCATCGCCGCGCTTTCGG-3′), synthesized by IDT (Coralville, USA), and cloned into vector pSpCAS9 (BB)-2A-Puro (Addgene, Cambridge, USA). Nuli-1 cells transfected with vector were selected using puromycin (Sigma, St. Louis, MA), and were isolated clonally. A_2B_AR knockout cells were confirmed by Western Blot analysis. Control cells were transfected with pSpCAS9 (BB)-2A-Puro.

### Western Blot

Cells lysates were prepared in RIPA buffer (Cell signaling, Danvers, USA). Equal amounts of protein lysates were separated by 8% sodium dodecyl sulfate-polyacrylamide gel electrophoresis (SDS-PAGE) and then transferred to polyvinylidene difluoride (PVDF) membranes. Membranes were blocked at room temperature for 1 h with 5% blotto (Santa Cruz, Dallas, USA) followed by exposure to Duox-1 antibody (1:500 dilution in 5% blotto) overnight at 4 °C. After washing with TBS plus 1% Tween-20, membranes were incubated with 1:5000 diluted goat anti rabbit IgG-HRP antibody for 1 h at room temperature. Membranes were incubated with Pierce Western Blotting Substrate (Pierce, Rockford, USA) and imaged. Antibody to GAPDH (Abcam, Cambridge, USA) was used as a loading control.

### Detection of Hydrogen Peroxide

Hydrogen peroxide (H_2_O_2_) concentrations were measured using a Hydrogen Peroxide Assay Kit (Abcam, Cambridge, USA), according to the manufacturer’s protocol.

### Measurement of Ca^2+^Release

Intracellular Ca^2+^ mobilization was assessed using a modification of procedures previously described by Tian *et al*.^31^. Briefly, Nuli-1 cells were grown at 1.5 × 10^4^ cells/cm^2^ on placenta collagen type VI coated LabTek glass chambers (Nunc, NY, USA). Cells were incubated with 5 μM Fluo-3 AM for 30 min at 37 °C, washed and placed on the stage of a Zeiss Confocal LSM 410 confocal microscope equipped with an Argon-Krypton Laser, 25 mW argon laser, 2% intensity (Thornwood, NJ). 0–20% CSE was manually added to a corner of the glass chamber and allowed to diffuse. Transient Ca^2+^ signal (measured as change in fluorescence, ΔF) was recorded.

### Statistics

Results are expressed as mean ± SE. Data were statistically analyzed using Student’s paired *t*-test followed by Tukey’s multiple-comparison test. Statistical differences among groups were determined using one-way ANOVA followed by Tukey’s multiple-comparison test (Graph-Pad Prism, version 5; Graph-Pad, San Diego, CA). Significance was assigned at P < 0.05.

## Additional Information

**How to cite this article:** Tian, Z. *et al*. Cigarette Smoke Impairs A_2A_ Adenosine Receptor Mediated Wound Repair through Up-regulation of Duox-1 Expression. *Sci. Rep.* **7**, 44405; doi: 10.1038/srep44405 (2017).

**Publisher's note:** Springer Nature remains neutral with regard to jurisdictional claims in published maps and institutional affiliations.

## Supplementary Material

Supplementary Information
