# Supplementary material for: Cigarette Smoke Impairs A2A Adenosine Receptor Mediated Wound Repair through Up-regulation of Duox-1 Expression
Source: Sci Rep. 2017 Mar 24;7:44405. doi: 10.1038/srep44405 (PMC5364501; doi:10.1038/srep44405)
Supplement: Supplementary Information [file srep44405-s1.pdf]

# Cigarette Smoke Impairs $A_{2A}$ Adenosine Receptor Mediated Wound Repair through Up-regulation of Duox-1 Expression

Zhi Tian[1], Hui Zhang[2], Jendayi Dixon[1], Nicole Traphagen[1], Todd A. Wyatt[2, 3,4], Kusum Kharbanda[4, 5], Samantha Simet Chadwick[2], Narasaiah Kolliputi[6], Diane S. Allen-Gipson[1, 2, 6]*

[1] Department of Pharmaceutical Sciences, College of Pharmacy, University of South Florida, Tampa FL; [2] Division of Pulmonary, Critical Care, Sleep and Allergy, Department of Internal Medicine, University of Nebraska Medical Center, Omaha, NE; [3] Department of Environmental, Agricultural, and Occupational Health, College of Public Health, University of Nebraska Medical Center, Omaha, NE; [4] Research Service, Omaha-Western Iowa Veterans Affairs Medical Center, Omaha, NE; [5] Division of Gastroenterology and Hepatology, Department of Internal Medicine, University of Nebraska Medical Center, NE; [6] Division of Allergy and Immunology, Department of Internal Medicine, College of Medicine, University of South Florida, Tampa FL.

Running head: Cigarette smoke generated hydrogen peroxide mediates Duox-1 up-regulation

*Address Correspondence to:

Diane S. Allen-Gipson. Ph.D.
Department of Pharmaceutical Sciences
College of Pharmacy
University of South Florida Health
12901 Bruce B. Downs Blvd, MDC 30
Tampa, Florida, 33612
Tel: 813-974-7225
Fax: 813-905-9885
Email: dallengi@health.usf.edu

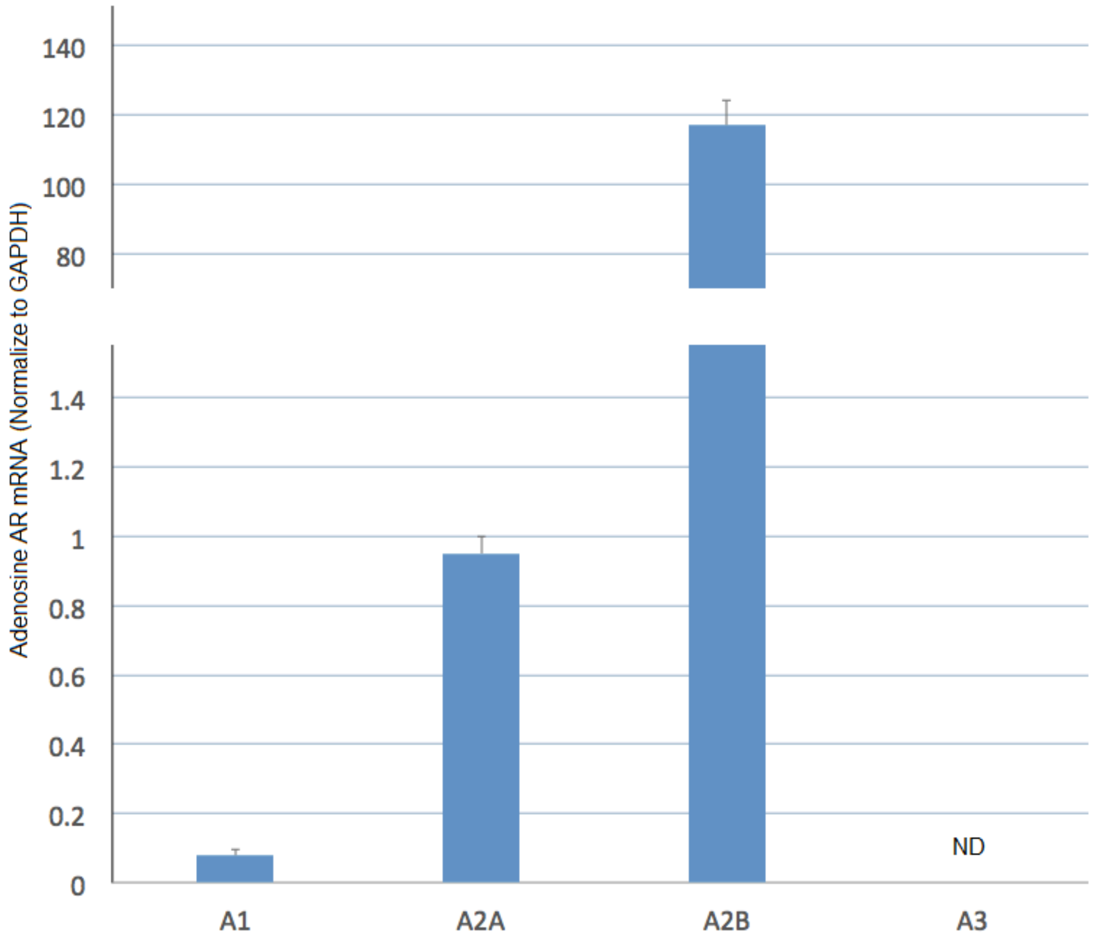


**Suppl. 1. Transcript levels of $A_1$AR, $A_{2A}$AR, $A_{2B}$AR, and $A_3$AR in normal Nuli-1 cells.**

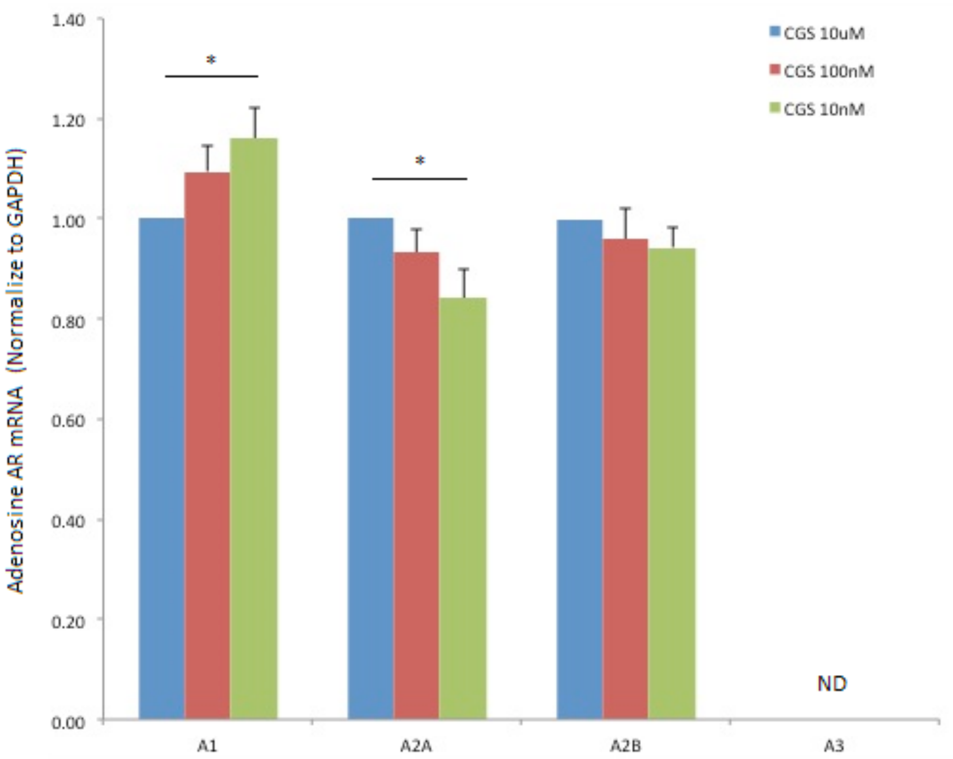


**Suppl. 2. Effect of CGS21680 on transcriptional levels of $A_1$AR, $A_{2A}$AR, $A_{2B}$AR, and $A_3$AR**. Expression levels of mRNA in Nuli-1 cells treated with CGS21680 for 24 h., * indicates significance value: $P<0.05$.
